# Conflit radio-ulnaire distal révélant un kyste anévrysmal osseux

**DOI:** 10.11604/pamj.2014.19.12.3038

**Published:** 2014-09-08

**Authors:** Aliou Amadou Dia

**Affiliations:** 1 Service de Radiologie de l'Hôpital Saint-Jean de Dieu, Thiès, Sénégal

**Keywords:** Conflit radio-ulnaire distal, kyste anévrysmal osseux, complexe triangulaire fibro-cartilagineux, Distal radioulnar conflict, aneurysmal bone cyst, triangular fibrocartilage complex

## Image en medicine

Le conflit radio-ulnaire distal résulte d'un pincement de l'articulation radio-ulnaire occasionnant un syndrome douloureux du poignet. Le conflit radio-ulnaire distal est le plus souvent d'origine dégénérative ou post-traumatique avec une atteinte du complexe triangulaire fibro-cartilagineux et un conflit ulno-lunaire associés par une inversion de l'index radio-ulnaire. Nous rapportons un cas de conflit de l'articulation radio-ulnaire distal chez une patiente de 35 ans, secrétaire de profession, consultant pour des douleurs chroniques du poignet gauche. Une radiographie du poignet en incidence de face objectivait une volumineuse lacune de l'extrémité inférieure du radius épiphyso-métaphysaire, bien limitée, aux contours irréguliers, d'aspects multi-cloisonné « soufflant l'os » sans rupture de la corticale osseuse du versant interne. Cette lésion expansive envahissait la membrane interosseuse et entrainait un conflit radio-ulnaire. La biopsie osseuse a été effectuée et revenait en faveur d'un kyste anévrysmal osseux. Le traitement chez notre patiente a consisté à un curetage des cavités kystiques associé à une greffe osseuse.

**Figure 1:**
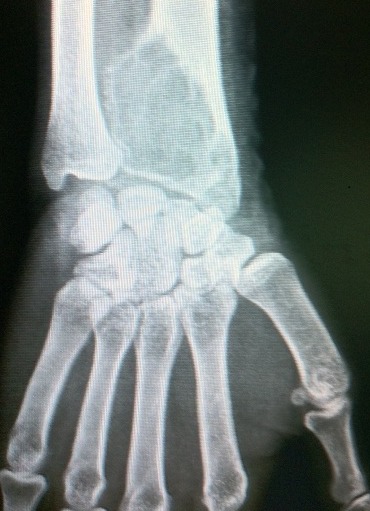
Radiographie du poignet de face montrant une volumineuse lacune multi-kystique de l'extrémité inférieure du radius, épiphyso-métaphysaire, bien limitée, « soufflant l'os » sans rupture de la corticale osseuse, envahissant la membrane interosseuse

